# Manipulating the Perceived Shape and Color of a Virtual Limb Can Modulate Pain Responses

**DOI:** 10.3390/jcm9020291

**Published:** 2020-01-21

**Authors:** Marta Matamala-Gomez, Birgit Nierula, Tony Donegan, Mel Slater, Maria V. Sanchez-Vives

**Affiliations:** 1 Institut d’Investigacions Biomèdiques August Pi i Sunyer (IDIBAPS), 08036 Barcelona, Spain; bnierula@gmail.com (B.N.); tonydonegan@gmail.com (T.D.); msanche3@clinic.cat (M.V.S.-V.); 2 Event-Lab, Department of Clinical Psychology and Psychobiology, Universitat de Barcelona, 08035 Barcelona, Spain; melslater@gmail.com; 3 Dipartamento di Scienze Umane per la Formazione ‘Ricardo Massa’, Università degli studi Milano-Bicocca, 20126 Milan, Italy; 4 Max Planck Institute for Human Cognitive and Brain Sciences, 04103 Leipzig, Germany; 5 Departament de Cognició, Desenvolupament i Psicologia de l’Educació, Facultat de Psicologia, Universitat de Barcelona, 08035 Barcelona, Spain; 6 Institució Catalana de Recerca i Estudis Avançats (ICREA), 08010 Barcelona, Spain

**Keywords:** virtual reality, pain perception, telescoped effect, amputee patients

## Abstract

Changes in body representation may affect pain perception. The effect of a distorted body image, such as the telescoping effect in amputee patients, on pain perception, is unclear. This study aimed to investigate whether distorting an embodied virtual arm in virtual reality (simulating the telescoping effect in amputees) modulated pain perception and anticipatory responses to pain in healthy participants. Twenty-seven right-handed participants were immersed in virtual reality and the virtual arm was shown with three different levels of distortion with a virtual threatening stimulus either approaching or contacting the virtual hand. We evaluated pain/discomfort ratings, ownership, and skin conductance responses (SCRs) after each condition. Viewing a distorted virtual arm enhances the SCR to a threatening event with respect to viewing a normal control arm, but when viewing a reddened-distorted virtual arm, SCR was comparatively reduced in response to the threat. There was a positive relationship between the level of ownership over the distorted and reddened-distorted virtual arms with the level of pain/discomfort, but not in the normal control arm. Contact with the threatening stimulus significantly enhances SCR and pain/discomfort, while reduced SCR and pain/discomfort were seen in the simulated-contact condition. These results provide further evidence of a bi-directional link between body image and pain perception.

## 1. Introduction

Immersive virtual reality (VR) technology has been repeatedly demonstrated to be an effective tool for modulating pain threshold perception in healthy subjects [1,2], and pain ratings in patients with chronic pain [3,4]. This is because multisensory signals, which can be integrated and manipulated in VR environments, influence our perception of pain, in part because nociceptive stimuli activate a wide network of cortical and subcortical areas in the brain, commonly known as the “pain matrix,” that are also implicated in the processing of sensory information [5].

Using VR, one can feel immersed (feeling inside of and being able to interact with the virtual world), and present (the subjective illusion of “being there,” when placed in the immersed virtual environment despite the knowledge that you are not there) [6] in a multisensory environment that is under the full control of the experimenter [7]. Furthermore, it is possible to induce, through VR, the illusion of owning a virtual body; this refers to the subjective illusion that a body or body part is one’s own, which again is facilitated by multisensory feedback such as synchronous visual and tactile stimulation, when the virtual and the real bodies are co-located at the same position [8]. The sense of ownership over a virtual body, or “embodiment,” is highly flexible regarding the visual aspect of the body, and it is possible to embody bodies that are quite different from those in real life [9,10]. The virtual body can therefore be designed with the morphological characteristics that the experimenter determines [11]. This allows the exploration of how the visual aspect of the body modulates pain perception. For example, Martini et al. [2] investigated the influence of skin color on pain perception when participants were embodied in a virtual body. They found that participants experienced pain threshold reduction when the virtual arm was represented in red compared to a “normal” or blue color. In addition, another study by the same group showed that virtual arm transparency decreases the pain threshold [12]. It is also possible to alter pain perception and anticipatory responses to pain in healthy subjects by modulating the morphological characteristics of the limb, such as showing a subject’s limb becoming smaller or bigger [13]. Moreover, we can reduce pain perception in a patient with chronic pain whose painful limb feels bigger than it really is by reducing the apparent size of their painful limb [14]. This top-down modulation of pain through modification of visual input reveals the potential of VR illusions as a treatment for pain. Additionally, instead of virtual body illusions, other studies have used VR to provide immersive virtual environments as a distractive pain strategy [15,16].

Even though VR is a potential tool for modulating the pain threshold through virtual body illusions in healthy subjects [1,2,12,17,18,19,20], this cannot be extrapolated to how these strategies are going to work in chronic pain patients. Indeed, there are some conflicting results about how bodily illusions can reduce chronic pain in clinical populations [21,22]. In this regard, a study conducted with thirteen amputee patients showed that, while phantom pain was reduced after four weeks of mirror therapy training in five amputee patients, eight amputee patients who reported telescopic distortion of the phantom limb (telescopic distortion refers to the feeling that the proximal portion of the amputated limb is missing or has shrunk with the more distal portion floating near, attached to, or ‘within’ the stump [23,24]), reported a gradual increase in phantom pain perception after the same mirror therapy training [22]. This difference may be due to the different cortical representations of the limb, a phenomenon seen not only in amputees but also in patients with complex regional pain syndrome and other chronic pain conditions [25,26,27,28,29]. The altered representation of the painful part of the body in the brain seems to play a key role in the development and maintenance of chronic pain, and therapies that attempt to reverse these changes have been partially successful, especially in amputee patients [30]. However, few studies have investigated how distorting a representation of a body part affects the sensation of pain. The present study aims to ascertain whether the illusion of ownership over a telescopically distorted virtual arm can modulate pain perception. To study pain, without delivering painful stimuli and avoiding the problems associated with sensitization/adaptation to repeated painful stimuli, we have used a paradigm of anticipatory responses to pain. Since the responses to a threatening stimulus start before skin contact [13], we investigated both the responses to pain when a threatening stimulus touched the virtual hand of the participant and the anticipatory responses to pain as a threatening stimulus approached the virtual hand. In order to deliver an unpleasant sensation to the participants, we used a vibration stimulus attached to the palm associated and triggered by a virtual needle as a threatening stimulus. For this, the skin conductance responses (SCRs), corresponding to the activation of the autonomic nervous system [31], of healthy subjects were recorded while they were embodied in a virtual body in several conditions in which the virtual arm, which was co-located with their real arm, had a normal or a distorted representation. Moreover, as was done in previous investigations [2,32], we wanted to investigate the effects of the redness of a colored telescopically distorted virtual arm on pain both when a threatening stimulus touched the virtual hand of the participant, and the anticipatory response to pain as a threatening stimulus approached the virtual hand.

The current study is a proof-of-concept study that may help us understand the mechanisms of visual distortion of body image, such as the telescoping effect in amputees (the distal part of the phantom limb perceived as shrinking within the stump), and its effect on pain responses.

## 2. Methods

### 2.1. Participants

Thirty right-handed, healthy subjects above 18 years of age participated in this study. Three subjects were excluded from further analysis due to an extremely high *z*-score (>± 2.5) in the SCR data, which led to a final sample size of 27 right-handed subjects (8 males and 19 females; mean age ± SD = 24.7 ± 1.1; mean Edinburgh scale ± SD = 68.7 ± 1.1). The following conditions were considered as inclusion criteria for participation in the study: normal or normal-when-corrected vision, the absence of neurological disorders, no history of chronic pain or other conditions interfering with pain sensitivity, no presence of epilepsy, no medication in use that changed attention to pain or general perception for 24 h before the experiment, and no pregnant women. The experiment was carried out in the installations of the Eventlab for Neuroscience and Technology Laboratory at the University of Barcelona/IDIBAPS. All participants gave written informed consent and received monetary compensation for their participation (12 €). The study was approved by the local ethics committee (Comité Ético de Investigación Clínica de la Corporación Sanitaria Hospital Clínic de Barcelona, HCB/2017/1068) and was carried out according to the Declaration of Helsinki [33].

### 2.2. Study Design

**Figure 1:**
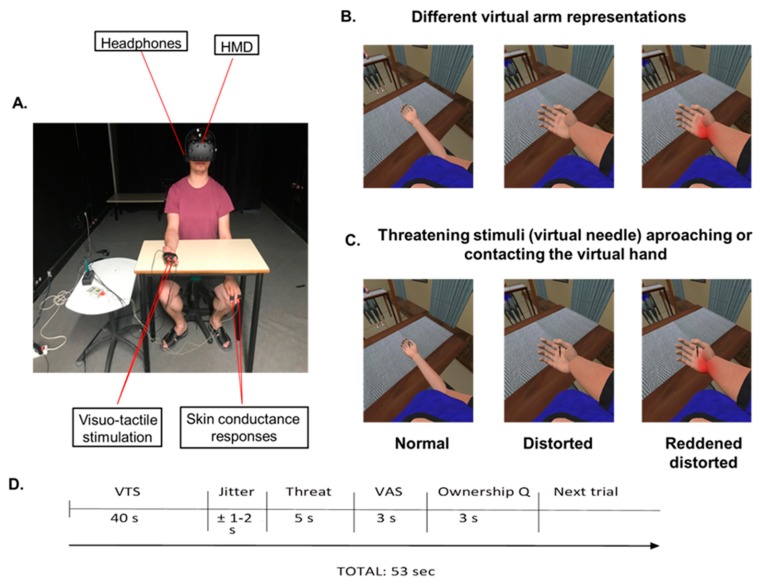
Experimental set-up and virtual arm and threatening stimulus conditions. (**A**) Participants wore a head-mounted display (HMD) that immersed them in a virtual environment. This allowed them to feel embodied in a virtual body, which they saw from a first-person perspective and which was co-located with their real body. Using headphones, participants heard the task instruction, “Pay attention to the right arm placed on the table, please,” before each visuo-tactile stimulus phase, which lasted 45 s. During the visuo-tactile stimulation, which was used to induce ownership over the virtual arm, virtual balls tapped the virtual fingers while participants felt, simultaneously, a tactile stimulation (vibration) on their real fingers. To record skin conductance responses after each threatening stimulus, two electrodes were attached to the index and ring fingers of the participants’ left hands. (**B**) Different virtual arm representations (virtual arm factor): normal representation, distorted representation (telescoped virtual arm), and reddened-distorted representation of the virtual arm. The distorted representation of the virtual forearm was shrinking within the virtual arm, as occurs with the telescoping effect in amputee patients. However, from a participant’s first-person perspective it seems bigger than the normal representation. (**C**) Threatening stimulus (virtual needle) in all three levels of the virtual arm factor. (**D**) Timeline of one experimental trial. Each experimental trial lasted around 53 s and was divided into four parts: First, participants were immersed in an immersive virtual reality (VR) environment in which the virtual arm could either be distorted, reddened and distorted, or in a normal position. To induce ownership over the virtual arm, they received 45 s of synchronous visuo-tactile stimulation. Second, after a jitter of 1–2 s, the threatening event appeared (a virtual needle) for 5 s. Immediately after the threatening event, the VAS appeared on the screen of the HMD. Finally, after the VAS was taken, a question related to ownership over the virtual arm appeared. VTS, visuo-tactile stimulation VAS, visual analogue scale; Ownership Q, ownership questionnaire.

In order to investigate whether observation of a telescopically distorted representation of the arm alters pain responses and anticipatory responses to pain in healthy subjects, participants completed one experimental session of 20 min. This study was a 3 × 2 within-subject experimental design. The experimental setup is shown in Figure 1A. In this study, there were two main factors. The first factor was Virtual Arm, with three different representations of the virtual arm: (1) the virtual arm represented in a normal position (Control); (2) the virtual arm represented in a distorted position, namely, a shortened virtual forearm shrinking within the arm (telescoped virtual arm) (Distorted); and (3) the virtual arm represented in a distorted position (telescoped virtual arm) and was red (Reddened-Distorted) (see Figure 1B). In all cases, the virtual arm was co-located with the real arm. The telescoped virtual arm representation was created following the graphical representation illustrating telescoping from the 5th edition of *Practical Management of Pain* [34]. In order to explore how these different representations of the virtual arm alter anticipatory responses to pain and pain responses itself, the second factor was *Threatening Stimulus Contact,* with two different types of threatening stimulus: (1) a tactile stimulus that contacted the skin (Real Contact); and (2) a stimulus that approached but did not touch the skin (Simulated Contact). For this, we used a vibrator attached to the palm of the hand of the participants to deliver visuo-tactile stimulations when the threatening stimulus (a virtual needle) contacted the palm of the virtual hand (see Figure 1C). Note that this is a tactile but non-nociceptive stimulus. The three virtual arm representations were combined with the two visually threatening stimulus contacts, resulting in six different conditions, each of which was presented three times. The order of the conditions was randomized among the subjects. Hence, each participant completed a total of 18 virtual arm and threatening stimulus exposures. After each exposure, participants had to indicate their level of sensory intensity (pain intensity) and affective magnitude (unpleasantness/discomfort), assessed using a visual analogue scale (VAS) [35] through a single request: “On a scale from 0 to 100, indicate the level of pain intensity/discomfort that you felt, please.” Further, after each exposure, participants had to indicate their level of ownership over the virtual arm [12]. Each stimulus exposure lasted 53 s (Figure 1D). After completing the virtual reality experiment, participants had to complete a questionnaire to evaluate their overall virtual reality experience [36]. In order to measure the electrodermal response when the threatening stimulus eventually touched the skin, and the anticipatory physiological response to an incoming threatening stimulus, the SCR was recorded following exposure to the threatening stimulus [13,37], which in the real contact condition touched the virtual hand and in the simulated contact condition simply approached the virtual hand without contacting it.

### 2.3. Apparatus

#### 2.3.1. Head-Mounted Display

We used a head-mounted display (HMD) (Rift Development Kit 2, Oculus, Menlo Park, CA, USA) with a resolution of 960 × 1080 pixels per eye and a nominal horizontal field of view of 100° displayed at 75 Hz to show the virtual environment, which was programmed in Unity 4.5.3 (Unity Technologies, San Francisco, CA, USA). The virtual body was configured to match the gender of the participants (female or male) and was taken from the Rocketbox library (Rocketbox Studios GmbH, Hannover, Germany). The virtual environment was the same during all conditions. Headphones were used in order to allow the participants to follow the task instructions during the experimental sessions (Figure 1A).

#### 2.3.2. Visuo-Tactile Stimulation

To increase the illusion of ownership over the virtual body, we used visuo-tactile stimulation. For this, we delivered tactile stimulation to the participants by using vibrators attached to the middle and index fingers, and to the palm of the right hand, that were controlled by Unity through an Arduino MEGA microcontroller board (Figure 1A). Each vibration had a duration of 1.0 s.

#### 2.3.3. Skin Conductance Responses

To record skin conductance, we attached two electrodes to the index and the ring fingers of the left hands of the participants (Figure 1A). The SCR was recorded at a sampling rate of 256 Hz, using a portable biosignal acquisition device (g.MOBIlab+, g.tec), while the recording and storage of the data was handled by a Simulink model in Matlab 2012b (The MathWorks, Inc., Natick, MA, USA).

### 2.4. Procedures

#### 2.4.1. Position of the Participants

Participants were seated on a chair with their right arm resting on the table and their left arm hidden under the table, resting on the left leg to ensure that participants only paid attention to their right arm during the experimental session. The right arm of the subject was placed within their field of view. The two vibrators attached to the dorsal distal phalanges of the right index and middle fingers were used in order to deliver visuo-tactile stimulations to induce the ownership illusion over the virtual arm. The other vibrator attached to the palm of the hand was used to deliver visuo-tactile stimulations when the threatening stimulus (the virtual needle) contacted the palm of the virtual hand. Through the HMD, participants were immersed in a virtual reality scenario in which they saw a virtual body from a first-person perspective co-located with their own real body [38]. They heard the task instructions through headphones throughout the experimental session, and their skin conductance was recorded with the two electrodes attached to their left index and ring fingers.

#### 2.4.2. Virtual Reality Scenario

Once participants donned the HMD, the room lights were turned off to allow the participants to be fully immersed in the virtual environment. At the beginning of the experimental session, participants were instructed to look around the virtual room, to describe what they saw, and to look down at their virtual body in order to habituate to the virtual scenario and the virtual body. The right virtual arm was always placed in the field of view of the participants with the palm of the hand facing up to the celling. Once the habituation phase was over, participants were asked to focus their attention on the right virtual arm. During the entire session and before the presentation of each condition, participants listened to the following verbal instruction through the headphones: “Pay attention to the right arm that is located on the table, please.” In order to induce the illusory ownership over the virtual body in each different representation of the virtual arm, each representation of the virtual arm included 40 s of visuo-tactile stimulation where participants saw a virtual ball tapping in random order their virtual right index and middle finger whilst feeling a spatiotemporal synchronous vibration on their real right index and middle finger, respectively [39]. During the experimental session, participants were exposed to six conditions in which three representations of the arm were combined with two types of stimulus contact.

#### 2.4.3. Pain Ratings and Ownership Measures

Pain ratings and measures of the degree of ownership over the virtual arm were taken for each of the six conditions to measure the effect of visual distortion on the sensory and anticipatory aspects of pain processing. Once each virtual trial was over, the screen of the HMD turned black and a VAS appeared with a voice instruction asking the participants to judge how strong the pain feels in order to assess the intensity of pain and how discomforting the threatening stimulus was perceived, on a scale ranging from 0 (not discomfort at all/minimum pain intensity) to 100 (worst discomfort/strongest pain intensity imaginable) [13]. Participants’ ratings were promptly annotated by the experimenter. After the VAS, a question related to ownership over the virtual arm in each representation appeared in the screen of the HMD, with a voice instruction asking the participant to judge their level of ownership over the virtual arm from −3 (totally disagree) to 3 (totally agree), with the following sentence: “I felt that the virtual arm was my arm” [36]. After the virtual reality exposure, the HMD and the headphones were removed and participants had to fill in a questionnaire concerning the overall virtual reality experience.

### 2.5. SCR Data Preprocessing

The peak-to-base response amplitude of skin conductance was used as an index of SCR [40,41,42]. For the assessment of SCRs, the difference between the maximum value detected in a 6 s post-stimulus time window and the baseline (3 s pre-stimulus) was computed, which is comparable with other studies in which a time frame of 1–5 s after stimulus onset was chosen [43,44,45]. Finally, we obtained the normalized maximum change of the SCR after each stimulus (virtual needle real contact/virtual needle simulated contact). The sample rate to extract SCR data was set to 256 Hz. The data were stored under Matlab and could be opened in the Matlab command windows with g.BSanalyze (gtec). Final SCR data were obtained by using a Matlab graphical user interface (GUI) for feature extraction of the skin conductance signal *(featextractiongui)* in Matlab2012b (The MathWorks, Inc. Natick, MA, USA). Event markers identifying each stimulus type were programmed to automatically register SCR when the stimulus—the virtual needle—contacted (real contact) or approached (simulated contact) the palm of the virtual hand.

### 2.6. Virtual Reality Experience Questionnaire

Once participants completed the whole VR exposure, they had to fill in a questionnaire related to their VR experience, answering the following statements from −3 (totally disagree) to +3 (totally agree) [36]:

Q1. During the experiment there were moments in which I felt that the virtual balls were touching my real fingers.
Q2. Although the virtual body did not seem to be physically my body, I felt that it could be my own body.
Q3. When I saw the virtual arm distorted, I felt that my own arm was distorted as well.
Q4. During each different representation of the virtual arm, I felt that if I moved my real arm the virtual arm would start moving too.
Q5. During the whole experimental session, I was able to focus my attention to the right arm.

### 2.7. Data Handling

All statistical tests were performed in Stata 13 (StataCorp LP, College Station, TX, USA). This was a mixed-effects design, with fixed-effects virtual arm (normal, distorted, reddened-distorted) and threat contact (real contact, simulated contact), and random effects over the individual subjects. We analyzed differences in pain ratings and in SCR across conditions with a multilevel mixed-effects linear regression test (the “mixed” function in Stata). Furthermore, in order to observe differences between virtual arm conditions, we ran a pairwise comparison with the Scheffe test for multiple comparisons. Moreover, in order to observe a possible relationship between ownership and pain ratings assessed with the VAS throughout the experiment, we used Spearman’s correlation test. In order to observe differences in ownership scores across conditions, we used multilevel mixed-effect ordered logistic regression test (the “meologit” function in Stata). Finally, in order to conduct mediation analyses, we used a seemingly unrelated regression test (the “sureg” function in Stata).

## 3. Results

### 3.1. Changes in Shape and Color of the Virtual Arm Modulate SCR after Threatening Stimulus Exposure

**Figure 2:**
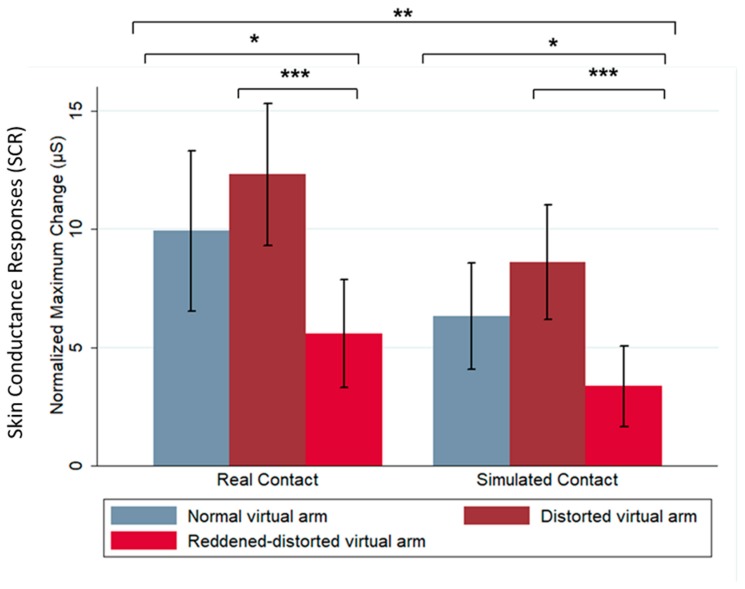
Skin conductance response (SCR) increased when the virtual needle contacted (real contact) the virtual hand in all three virtual arm conditions, while the reddened-distorted virtual arm showed a comparatively decreased SCR in both real and simulated contact of the threatening stimulus. Difference in SCR after the threatening stimulus contacted (real contact) or approached (simulated contact) the virtual hand in all three virtual arm conditions. Bars show mean change in SCR and error bars indicate 95% confidence interval. * *p* < 0.05, ** *p* < 0.01, *** *p* < 0.001.

Our results show that changes in shape and color of the virtual arm modulated SCR after a threatening stimulus exposure. In this case, the SCR was taken as a proxy of pain responses, as has been established in previous work by Romano and Maravita [13]. Specifically, we observed a higher SCR when the virtual arm was distorted (telescoped virtual arm) compared with the normal virtual arm (*z* = −2.93, *p* = 0.003); we did not find a significant relationship between the shape changes of the virtual arm and SCR, but did find a significant relationship between color changes and SCR. The results show that the red color in the reddened-distorted virtual arm increase anticipatory pain responses, showing a significant difference between normal (*z* = −2.93, *p* = 0.014) and distorted (*z* = −4.79, *p* < 0.0001) virtual arm conditions. Furthermore, in relation to the dependency of the SCR on the threatening stimulus contact, our results showed a significant decrease in SCR when the threatening stimulus approached but did not contact the virtual hand (simulated contact), compared to the real contact condition *(z* = −3.08, *p* = 0.002) (Figure 2). Interestingly, the proportions between SCR in normal virtual arm, those of distorted and reddened-distorted arms, were the same both for the real contact and the simulated contact; however, the absolute values were smaller in the simulated contact. Table 1 summarizes SCR values and *p*-values for all of the experimental conditions.

### 3.2. Distortion of the Virtual Arm Increase Pain Ratings (VAS) after Threatening Stimulus Exposure

**Figure 3:**
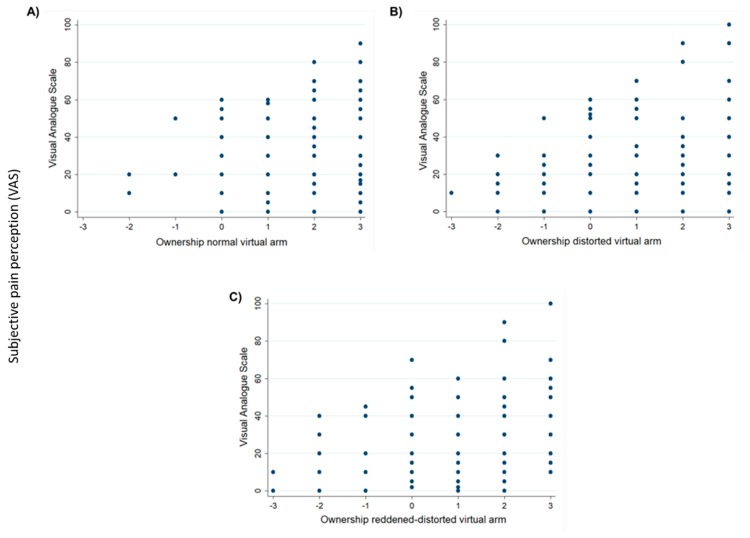
Pain ratings increase with the distortion of the embodied virtual arm. The relationship between pain ratings and ownership levels under the (**A**) normal virtual arm condition (no relationship); (**B**) the distorted virtual arm condition; and (**C**) the reddened-distorted virtual arm condition.

The data obtained in our study show a positive relationship between the level of ownership over the distorted (*r_s_* = 0.226, *p* < 0.01) and reddened-distorted (*r_s_* = 0.225, *p* < 0.01) virtual arms with the level of pain/discomfort assessed using the VAS. Nevertheless, this positive relationship was not found after the normal virtual arm exposure (*r_s_* = 0023, *p* = 0.767) (Figure 3A–C). Thus, in the distorted virtual arm conditions, we found that the higher the level of ownership of the distorted and reddened-distorted virtual arm, the higher the pain/discomfort perception. Furthermore, in agreement with the above results in which we observed a significant difference when the threatening stimulus contacted or approached the virtual arm, again our results show a significant difference in pain/discomfort perception when the threatening stimulus contacted or approached the virtual arm. In particular, we found lower VAS scores when the threatening stimulus approached the virtual hand, but not when it contacted the virtual hand (multilevel effects mixed effects *z* = −7.00, *p* < 0.001).

### 3.3. Dependency of Ownership on Shape and Color Changes of the Virtual Arm after Threatening Stimulus Exposure

**Figure 4:**
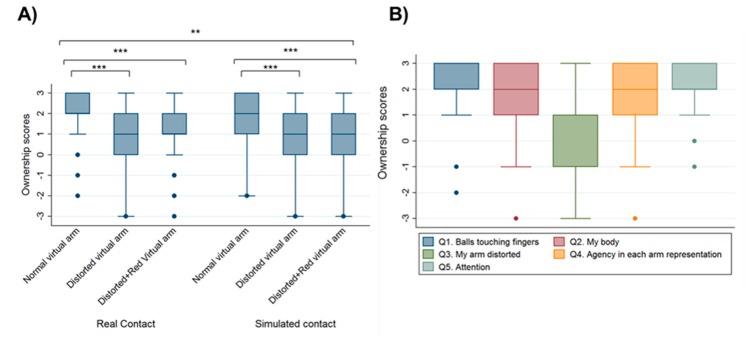
Ownership levels increase with the normal representation of the virtual arm. (**A**) Ownership question ratings after each virtual reality condition exposure show higher ownership scores in the normal virtual arm representation compared with the distorted and reddened-distorted conditions, in both real and simulated contact of the threatening stimulus. (**B**) Questionnaire ratings after the whole virtual reality exposure show that although participants reported high levels of agency and ownership of the virtual body, ownership scores decreased with the distorted virtual arm representation. Boxplots show medians (horizontal lines), interquartile ranges (IQR; boxes), data outside 1.5 × IQR (whiskers), and outliers (o). ** *p* < 0.01, *** *p* < 0.001.

The reported levels of ownership show a statistically significant difference between the different representations of the virtual arm, ownership being higher with the normal representation of the virtual arm. Indeed, we found a statistically significant difference in ownership between the normal representation of the virtual arm and both the distorted (*z* = −9.16, *p* < 0.001) and reddened-distorted virtual arm (*z* = −8.92, *p* < 0.001). In addition, contact of the threatening stimulus with the virtual hand significantly increased the level of ownership in all three virtual arm representations compared with simulated contact of the threatening stimulus (*z* = −2.30, *p* = 0.021) (Figure 4A). Table 2 summarizes ownership values and *p*-values for all of the experimental conditions.

From the results obtained in the final VR questionnaire that participants had to fill in after the VR experience, we found that participants reported high levels of ownership (Q1, Q2) and agency (Q4) of the virtual body and virtual arm. However, we also observed low scores in ownership levels with the distorted virtual arm representation (Q3). Finally, in Q5, which was related to the level of attention towards the virtual arm, participants reported high attention levels of the virtual arm throughout the whole VR exposure (Figure 4B).

We found that ownership (ownership scoring collected during the experimental sessions) mediates differences in SCR, but not subjective pain ratings (VAS), between the different virtual arm conditions. Specifically, we found a significant relationship between the independent factor virtual arm and the mediation variable ownership (*z* = −5.39, *p* < 0.001). Further, we also found a significant relationship between the SCR (dependent variable) and ownership (*z* = −2.91, *p* = 0.004), and between the SCR and the virtual arm factor (*z* = −3.34, *p* = 0.001). We calculated the mediation effect (ME) by the following calculation: ME = regression coefficient between SCR and ownership (*r_s_* = −1.13) × regression coefficient between SCR and virtual arm (*r_s_* = −0.42). The ME of ownership in SCR between the different virtual arm conditions is 0.472. Although we did not find a relationship between the subjective pain ratings (VAS), as a second dependent variable, and the virtual arm factor (*z* = 0.90, *p* = 0.367), we found a significant relationship between VAS and ownership (*z* = 5.31, *p* < 0.001). Nevertheless, no mediation effects of ownership can be reported in subjective pain responses ratings (VAS) between different virtual arm conditions.

## 4. Discussion

Through immersive virtual reality, we can induce the sense of owning a virtual arm by using a congruent multisensory correlation between the real and the virtual arm [8,46]. It is known that changes in the color and shape of a virtual arm (that is co-located with the real one) modulate pain threshold in healthy subjects (see [47] for a review). Furthermore, there is some evidence demonstrating that a distorted representation in the brain of the painful part of the body is associated with increased pain perception in chronic pain patients [48,49]. Along these lines, some studies have investigated the role of the representation of the painful part of the body in the brain, such as the telescopic effect in amputees, on pain perception [25,50]. The study by Makin and colleagues [25] highlighted the importance of the preserved representation of the amputated limb in the area of the brain where the former hand was represented [51] as a key factor involved in chronic phantom pain. In their study, the authors suggested that this preserved representation could activate phantom chronic pain through top-down (central to peripheral nervous system) mechanisms. In line with this study, Bultitude and Rafal [26] suggested that the pain in patients with complex regional pain syndrome is a consequence of a distorted representation of the affected limb in the brain. Furthermore, some patients with chronic pain report that the mental representation of their affected body part is somehow distorted in size or posture, or even absent entirely [27,28,29], which may be associated with a distorted cortical representation of the limb. While early investigations found that visual feedback techniques such as mirror therapy or virtual reality could reduce pain perception in amputee patients [30,52,53], it has also been shown that mirror therapy can exacerbate pain in those amputees that experience the telescopic effect [22].

Here, we conducted a proof-of-concept study in healthy subjects in order to better understand the mechanisms of pain/discomfort in amputee patients that suffer from the telescoping effect. Although the mechanisms for acute and chronic pain are different [54], and amputees and healthy individuals are likely to have significantly different cortical representation of their body [55], this study can contribute to the understanding of the effects of visual distortion of the body on pain. We observed that multisensory integration interventions using VR could be used to manipulate the shape and color of the virtual body representation in order to modulate pain perception and anticipatory responses to pain in healthy subjects. We conclude that autonomic responses (SCR) that act as proxies of pain responses are enhanced when there is a virtual threat (virtual needle) that approaches or eventually touches a virtual arm that feels a part of one’s own body. This paradigm can be used in order to explore how changes in the morphological or functional characteristics of the represented virtual body can modulate pain responses while avoiding the problems derived from repetitive painful stimulation, such as sensitization and adaptation. Although existing studies have demonstrated that visual illusions in which arm size is increased or decreased can affect pain perception in both healthy and clinical populations [4,13,56], this is the first study to investigate how being embodied in a virtual body with a distorted virtual arm (simulating the telescopic effect in amputees), affects subjective (pain scores in VAS) and the related physiological responses (SCR) to a threatening event in healthy subjects.

First, regarding the virtual arm factor, we found that being embodied in a distorted virtual arm enhances SCR and pain/discomfort ratings to the threatening event compared with the normal virtual arm condition; these results are in line with later investigations [13] in which the authors found higher SCR while observing a visually magnified hand. Moreover, other studies also found that visual enlargement of the hand being viewed enhanced analgesia by increasing heat-pain thresholds, compared with the visual reduction of the viewed hand [57]. However, the opposite effect was found in clinical populations with chronic pain, in which the visual enlargement of the affected hand increased pain perception compared with the visual reduction of the affected hand [14]. Furthermore, in the former study [57], they also found that visual enlargement of the viewed hand enhanced analgesia by increasing heat-pain thresholds, while visual reduction of the viewed hand reduced analgesia. However, the opposite effect was found in clinical populations with chronic pain, in which the visual enlargement of the affected hand increased pain perception compared with visual reduction of the affected hand [14]. Furthermore, in another study, a negative body image associated with an injured-appearing hand (induced using the rubber hand illusion paradigm) reduced the pain threshold in healthy subjects, but no such increase was seen with a visually distorted (stretched) rubber hand [58]. One possible explanation for the difference is that the distorted rubber hand was not exactly co-located with the real hand of the participant, diminishing the sense of ownership of the rubber hand, as surmised by Nierula and co-authors [17].

In a previous study, Romano and Maravita [13] found that visual distortion of the hand by giving a visual minifying hand feedback increased SCR in healthy subjects, whilst a magnified visual feedback of the hand decreased SCR, which suggests that the visual size increase enhances the cognitive, anticipatory component of pain processing. In our study we also found that SCR was lower in the reddened-distorted virtual arm condition compared with normal and distorted virtual arm conditions. In line with the study from Romano and Maravita [13], we speculate that in our study the red color in the reddened-distorted virtual arm enhanced the cognitive, anticipatory component of pain processing and decreased the SCR to the subsequent threatening stimulus. In fact, it is known that by observing a reddened embodied virtual arm, we can reduce the pain threshold in healthy subjects [2]. Hence, as participants were observing the reddened virtual arm 45 s before the threatening stimulus appeared (visuo-tactile stimulation phase) at the beginning of each condition, we can speculate that the red color caused an anticipatory pain response, resulting in a reduction in SCR following the threatening stimulus being in contact or simulated contact with the virtual arm. This effect could be induced via several mechanisms: on one hand, anticipatory responses to pain could induce the activation of endogenous descending analgesic neural pathways [59], involving subcortical reticular structures that target the dorsal horns of the spinal cord grey matter, reducing afferent noxious signals. This interpretation could be explained by the principle of diffuse noxious inhibitory control, by which a noxious stimulus decreases the response (or increases the threshold) to a subsequent painful stimulus (see [60] for a review). On the other hand, the expectation of the incoming painful stimulus can induce a pre-activation of early somatosensory regions with a subsequently decreased response when the signal from the noxious stimulus arrives at the cortex [61]. Moreover, it is also known that the more certainty regarding a painful stimulus that modulates the expectancy about pain perception, the more the recruitment of attentional resources to the ascending nociceptive input [62]. Hence, one may hypothesize that changes in shape and color of an embodied virtual arm may modulate both physiological and subjective measures of pain responses.

Secondly, in our study we found a generally increased SCR and subjective measure of pain perception when the threatening stimulus touched the virtual hand, and a generally reduced SCR when the threatening stimulus approached but did not touch the virtual hand in all three virtual arm conditions. Our findings differ from those shown in the study by Romano and Maravita [13], in which they observed a reduced SCR with real contact noxious stimuli while observing a magnified hand was associated with increased anticipatory SCR when the noxious stimuli approached the skin without touching it. However, they also observed a general increase in SCR for both real and simulated contact while observing a shrunken vision of the hand. In our study we did not observe such differences in SCR between the different virtual arm conditions. One explanation for this could be the synchronous visuo-tactile stimulation that participants received in their real hand once the threatening stimulus touched the virtual hand. It is well known that through synchronous visuo-tactile stimulation we may induce a sense of ownership over a fake limb, as in the “rubber hand illusion” study [63], over a whole fake body [64] and over a virtual arm [8]. Therefore, we postulate that the fact of receiving tactile stimulation (vibration) at the same place of the real hand, at the same time that the threatening stimulus touched the embodied virtual hand, enhanced the feeling of the virtual needle hurting the virtual arm, thereby enhancing pain perception. Finally, participants experienced higher levels of ownership with the normal virtual arm condition either when the virtual needle touched or approached the virtual arm. Nevertheless, participants reported higher levels of ownership after the normal virtual arm representation in the real contact of the threatening event condition. Once again, this could be explained by the synchronous visuo-tactile stimulation that is induced when the virtual needle touches the virtual hand, which may enhance the sense of ownership over the virtual body [8]. Then, one may postulate that the observed results in the subjective pain responses and physiological responses to the threatening event may be related to the lower ownership responses towards the distorted (telescoped) virtual arm condition.

Finally, mediation effects of ownership ratings in SCR are open to several interpretations. First, this effect confirms that being embodied in a virtual body by providing a virtual body illusion can modulate physiological responses, as has been demonstrated previously [65,66,67]. Further, modulation of the morphological characteristics of the virtual body modulates physiological responses [68]. Our results show that changing the morphological characteristics of an embodied virtual body while being in a painful or threatening situation may modulate the physiological responses associated with pain, and these can be mediated by the feeling of ownership of the virtual body. While the use of embodiment to change body representations for pain relief has been already discussed (see [47] for a review), no mediation effects of ownership were found for the subjective pain measure (VAS ratings). One explanation of this may be because of the lower ownership ratings, compared to the normal virtual arm representation, obtained in the distorted and in the reddened-distorted virtual arm conditions. Although participants knew that the distorted virtual arm was not their real arm, it still influenced physiological responses of the participants, as has been demonstrated in previous studies [2,18,69]. Hence, even though participants felt less ownership in the distorted virtual arm conditions, physiological responses to pain were still modulated, highlighting the powerful effect of virtual embodiment in modulating body representations and their consequent impact on the modulation of physiological responses of pain responses.

## 5. Limitations

The present study shows a limitation regarding an unbalance in the participants’ genders. It is known that females and males perceive pain differently [70], so although in this study no experimental pain was induced, the threatening event and the distortion of the virtual arm could activate both pain and anticipatory pain responses.

## 6. Conclusions

The present study investigated the influence of telescoping effects on the perception of pain by inducing such limb distortions in healthy people through VR. Our findings are in line with other studies in which the authors demonstrated that healthy subjects could experience the telescoped effect and the associated telescoping sensations (pain/discomfort) normally experienced by amputee patients through full virtual body illusions. Further, the results of this study demonstrate that the link between body image and pain responses is bi-directional, showing a top-down effect of body image on pain. This bi-directional link has also been reported in other studies [22,25,50,71,72]. Furthermore, our results reinforce the importance of tackling body image distortions when trying to reduce pain responses in chronic pain patients, especially in amputee patients with a telescopic limb sensation, suggesting that VR could be a powerful tool for modulating pain responses by changing the representation of the telescoped limb.

## Figures and Tables

**Table 1:** Values in terms of means, standard error (SE), and *p*-values indicating mean differences between the different experimental conditions.

Experimental Variable	Virtual Arm			Threat Contact	
	Normal Virtual Arm (1)	Distorted Virtual Arm (2)	Reddened-Distorted Virtual Arm (3)	Real Contact (1)	Simulated Contact (2)
Mean	8.13	10.43	4.49	9.27	6.17
SE	13.20	12.63	9.25	13.60	10.09
*p*-value	0.014	<0.001			<0.001
Conditions	3 vs. 1	3 vs. 2			2 vs. 1

**Table 2:** Scoring values in terms of mean, standard error (SE) and *p*-values for all the different experimental conditions.

Experimental Variable	Virtual Arm			Threat Contact	
	Normal Virtual Arm (1)	Distorted Virtual Arm (2)	Reddened-Distorted Virtual Arm (3)	Real Contact (1)	Simulated Contact (2)
Mean	1.89	1.03	1.05	1.42	1.38
SE	1.13	1.49	1.50	1.23	1.49
*p*-value		<0.001	<0.001		0.021
Condition		2 vs. 1	3 vs. 1		2 vs. 1
